# Overall drug treatment of idiopathic pulmonary fibrosis patients from national registries – a real-world study from Finland

**DOI:** 10.1186/s12890-023-02630-1

**Published:** 2023-09-30

**Authors:** Jaana Kaunisto, Eija-Riitta Salomaa, Mari Koivisto, Marjukka Myllärniemi

**Affiliations:** 1 Department of Pulmonary Diseases, Division of Medicine, Turku University Hospital, Turku, Finland; 2 Department of Pulmonary Diseases and Clinical Allergology, University of Turku, Turku, Finland; 3 Department of Biostatistics, University of Turku, Turku, Finland; 4 Heart and Lung Center, Helsinki University Hospital, Helsinki, Finland

**Keywords:** Idiopathic pulmonary fibrosis, Antifibrotic therapy, Real-world data

## Abstract

**Background:**

Currently, two disease-modifying antifibrotic drugs are indicated for the treatment of idiopathic pulmonary fibrosis. The objective of this study was to analyse antifibrotic and overall prescription medication use of IPF patients in the real world.

**Methods:**

Data was collected from the FinnishIPF registry and the Registry of the Social Insurance Institution of Finland (SII). Purchases of all prescription medicines were assessed. The frequency, the initiation interval, the duration, and the breaks of the antifibrotic treatments were defined. The association between the prescription of antifibrotic therapy and different patient-related clinical parameters was studied. Accordingly, the relationships between the delay in starting therapy and patient-related variables were analysed.

**Results:**

Of the 263 IPF patients, 132 (50.2%) had started antifibrotic treatment during the study period 2011–2018. The mean interval from the diagnosis to the first purchase was 367 (SD 429) days. The antifibrotic drug was switched in 14% of patients. Discontinuation of therapy occurred most commonly during the first year of the treatment. The one-year persistence was 77.1% for pirfenidone and 78.9% for nintedanib. A tendency of treating patients under 75 years was noticed. Low forced vital capacity predicted earlier initiation of medication.

**Conclusions:**

The initiation of antifibrotics after diagnosis was slow, probably due to reimbursement limitations. Younger age at diagnosis affected treatment initiation although it is unknown which patients benefit most from these medications. The reasons for discontinuation of the antifibrotic therapy during the first year should be a focus in clinical work and further studies.

## Introduction

The 2010s were pivotal in terms of the diagnostics and treatment of idiopathic pulmonary fibrosis (IPF). After the launch of two disease-modifying antifibrotic medications, the dismal prognosis of IPF has promisingly changed for the better. In randomized control trials (RCT) pirfenidone and nintedanib have been shown to slow down the disease progression [1–3]. In pooled analyses and meta-analyses both antifibrotics seem to reduce mortality [4, 5]. In RCTs, patients are carefully selected, and studies are implemented in a controlled environment, the results are not fully generalizable to the real world. It is acknowledged that real-world data (RWD) are needed to complement the data from RCTs.

Early initiation of the antifibrotic treatment has been emphasized as the disease course is unpredictable and the prognosis is poor. Based on the results of the European survey 2016 [6], the “watch and wait” approach was often taken by physicians and patients, especially in mild and stable cases. In daily practice, there are several factors that influence the response to the medication. Additional data is needed to evaluate treatment implementations in a real-world setting.

IPF is associated with several comorbidities [7, 8] such as cardiovascular disease, gastroesophageal reflux, and chronic obstructive pulmonary disease. Multimorbidity often leads to the use of multiple medications [9]. To our knowledge, there are only a few RWD studies on the overall prescription medication use of IPF patients [10].

The aim of this study was to assess the use of antifibrotics and simultaneously prescribed medications based on observed drug purchases from the pharmacies by using our national drug reimbursement and purchase registry. Treatment delays, duration, and discontinuation frequency of the antifibrotic therapies were calculated. The relationship between the decision to initiate the antifibrotic treatment and different clinical variables was analyzed. Moreover, the associations between earlier initiation (within a year from the diagnosis) and clinical variables were studied.

## Material and methods

### FinnishIPF registry

The FinnishIPF registry is a nationwide registry collecting comprehensive longitudinal data of IPF patients [11] and implemented in all respiratory clinics across Finland. The registry also includes patients diagnosed before it was launched in 2011. The registry relies on a web-based platform (Granitics Unify Med, Granitic Ltd, Espoo, Finland). To be eligible for inclusion in the registry, the patient is diagnosed with IPF in accordance with international guidelines [12, 13]. Informed consent is required. Data entries are made by the study nurse or registry coordinator at each site. Diagnostic and follow-up information are collected from local electronic hospital records. The FinnishIPF registry currently contains clinical data of over 900 IPF patients.

### Registry of Social Insurance Institution 

Most of the medicines prescribed for illnesses in Finland are reimbursed by Social Insurance Institution (SII). Practically all prescription medications are reimbursed in Finland. SII holds statistics on the use of reimbursed medications. Annually each patient pays a maximum 592.16 € (in 2022) for the reimbursed medications and thereafter the prescribed medicines cost only 2.50 euros per purchase. Pirfenidone and nintedanib have been reimbursable in Finland since 1.6.2013 and 2015, respectively. The spirometric criterion for reimbursement was FVC 50–80% predicted from 1.6.2013. Currently (since 1.11.2015) the criterion is FVC % predicted is 50–90% at the time of application.

### Study subjects

Patients (*N* = 263) that consented to share both registry data (FinnishIPF and SII data) were included. Hereby inclusion criteria were: 1) confirmed IPF diagnosis and 2) consent to use both above-mentioned registries. A significant number of patients did not reply to the request to share SII registry data, thus lowering the number of participants of this study.

Data on medication use consisted of the date of the purchase and the number of packages of reimbursed medicines. The purchases were collected from the beginning of 2011 until the end of 2018. The number of concomitant medications (other than antifibrotic) was calculated for each patient by considering all different products purchased 120 days before and after the date of the diagnosis. Medication persistence was defined from the date of initiation to the date of discontinuation of the therapy (no purchases within 60 days).

For the analyses concerning therapeutic delay, the study population was limited to patients diagnosed on 1.6.2013 or after, since then antifibrotics have been available and reimbursable in Finland. Treatment delay was determined as an interval between the date of diagnosis and the date of the first purchase of antifibrotic medicine.

## Analysis

Continuous variables having a normal distribution were summarized with mean and standard deviation (SD), and those not having a normal distribution were summarized with median and lower quartiles (Q1) and upper quartiles (Q3). Categorical variables were summarized with counts (n) and percentages. Background variables were compared between groups using a two-sample t-test or Wilcoxon rank sum test and for categorical variables, a Chi-Squared test or Fisher’s exact test was used.

The number of concomitant medications (other than antifibrotic) was calculated for each patient by considering all different drug purchases 120 days before and after the date of diagnosis.

Univariate associations between the outcome variable (initiation of the antifibrotic medication) and study variables (age, gender, FVC %, DLCO/VA, and number of other medications) were studied using logistic regression analysis. Age and number of other medications were categorized in three categories (age 50–65, 66–75 and over 75 years and 0–1 medication, 2–4 medications and 5 or more medications), FVC % and DLCO/VA were categorized in two categories (75% or less and over 75% and 55 or less and over 55 respectively). The significant variables (age, FVC %, DLCO/VA) in the univariate analysis were included in a multiple logistic regression model. In addition, a subgroup analysis was conducted using the outcome variable “the delay of initiation of antifibrotic treatment (≤ 1 year vs > 1 year)” and only including patients that were diagnosed 1.6.2013 or later. The Kaplan–Meier method was used with the cumulative discontinuation curve.

Results are presented with odds ratios (OR) together with 95% Confidence intervals (CI).

All statistical tests were performed as 2-sided, with a significance level set at 0.05. Statistical analyses were carried out using SAS for Windows version 9.4 (SAS Institute Inc., Cary, NC, USA).

## Results

### Results of the whole study population (*N* = 263)

**Fig. 1:**
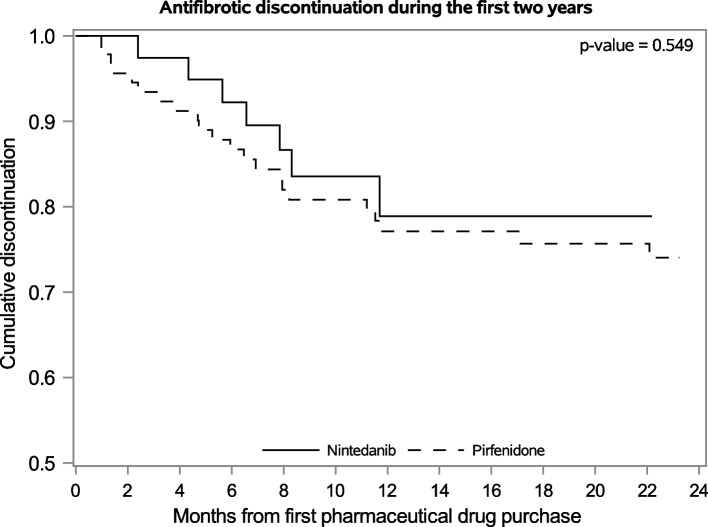
Persistence curve for pirfenidone and nintedanib. Persistence during the first two years

Of the 263 patients, 132/50.2% had started antifibrotic treatment – pirfenidone (92 patients) or nintedanib (40 patients). At diagnosis, the mean age was 70.4 (SD 8.8) years. 69.2% of the patients were men. 37.7% of patients were never smokers. The baseline characteristics of the study population are presented in more detail in Table 1. The mean treatment duration was 763 days (range 41–2004 days). Antifibrotic agents were switched in 18 patients (13.6%). Pirfenidone was switched to nintedanib in 13 patients, and in 1 case it was switched back. Nintedanib was switched to pirfenidone in six patients. According to the dates of purchases, breaks in the antifibrotic treatment were observed. The frequencies of different treatment breaks are presented in Table 2. Discontinuation of the therapies was most common during the first year. Treatment persistence with the antifibrotic therapies is visualized by the Kaplan–Meier curve, Fig. 1. The one-year persistence was 77.1% for pirfenidone and 78.9% nintedanib.

**Table 1:** Baseline characteristics of the study population. Data are presented as mean ± sd unless otherwise stated

Variable	All patients (*N* = 263)	Patients with antifibrotic (*N *= 132)	Patients without treatment (*N* = 131)	*p*-value
Age at diagnosis(years)	70.4 ± 8.8	68.3 ± 7.8	72.5 ± 9.3	0.0002
50–65 years N (%)	47 (19.8%)	31 (27%)	16 (13.1%)	< 0.0001
66–75 years N (%)	107 (45.2%)	60 (52.2%)	47 (38.5%)	
> 75 years N (%)	83 (35.0%)	24 (20.9%)	59 (48.4%)	
Gender				0.21
Male N (%)	182 (69.2%)	96(72.7%)	86 (65.7%)	
Female N (%)	81 (30.8%)	36 (27.3%)	45 (34.4%)	
BMI (kg/m^2^) median	27.8 (25.5,30.8)	28.0 (25.3–30.9)	27.8 (25.6–30.3)	0.91
FVC (% of predicted)	83.0 ± 17.4	77.3 ± 14.8	88.1 ± 17.9	< 0.001
FVC ≤ 75% N (%)	69 (33.7%)	42 (43.3%) 55 (56.7%)	27 (25%)	0.0056
FVC > 75% N (%)	136 (66.3%)		81 (75%)	
DLCO/VA (% of predicted)	62.2 ± 14.7	59.5 ± 14.2	64.3 ± 14.9	0.022
DLCO/VA ≤ 55 N (%)	66 (33.7%)	36(40.9%)	30(27.8%)	0.053
DLCO/VA > 55 N (%)	130 (66.3%)	52 (59.1%)	78 (72.2%)	
Smoking at diagnosis				0.93
Never smoker N (%)	96 (37.7%)	46 (36.5%)	50 (38.8%)	
Current smoker N (%)	20 (7.8%)	10 (7.9%)	10 (7.8%)	
Ex-smoker N (%)	139 (54.5%)	70 (55.6%)	69 (53.5%)	
Number of medications at diagnosis (other than antifibrotic) median	5(1,9)	5 (1,9)	6.0 (2.0,9.0)	0.65
0–1 medications N (%)	66 (25.1%)	34 (25.8%)	32 (24.4%)	0.90
2–4 medications N (%)	40 (15.2%)	21 (15.9%)	19 (14.5%)	
≥ 5 medications N (%)	157 (59.7%)	77 (58.3%)	80 (61.1%)

**Table 2:** Treatment breaks among antifibrotic users (*N* = 130, missing 2)

Break (days)	Frequency, N (%)
≥ 45	36 (27.7%)
≥ 90	19 (14.6%)
≥ 120	14 (10.8%)
≥ 180	11 (8.5%)

**Fig. 2:**
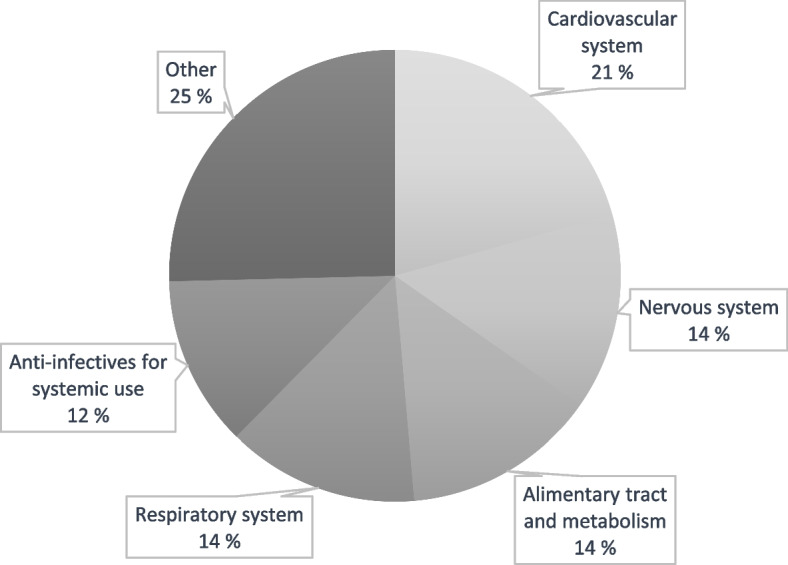
Most prescribed co-medications among antifibrotic users according to therapy areas (ATC-code)

At diagnosis, 59.7% of patients had five or more simultaneous medications. Most prescribed co-medications according to the therapy areas (ATC-code) are presented in Table 3 and Fig. 2. Cardiovascular medications were the most common (21% of patients). This group consists also of cholesterol-lowering medicines. 14% used medication classified as nervous system medications. This group includes painkillers, such as paracetamol and paracetamol-codeine, which is often used for cough in Finland, and sleeping pills. 14% used alimentary tract drugs including proton pump inhibitors and medicines for constipation. Also, 14% used respiratory medicines such as bronchodilating and combination inhalers.

**Table 3:** Five most common therapy areas and medications in IPF patients according to ATC classification are listed in descending order by prescription frequency

Therapy area	Most prescriped medication
Cardiovascular system (ATC class A)	Bisoprolol Simvastatin Amlodipin Atorvastatin
Nervous system (ATC class N)	Paracetamol Paracetamol + codeine Zopiclone Oxycodone
Alimentary tract and metabolism (ATC-class A)	Pantoprazol Metformin Esomeprazol Ispaghula husk
Respiratory system (ATC-class R)	Salbutamol Tiotropium bromide Fluticasone propionate + azelastine Ciclesonide Monometasone furoate Fluticasone furoate
Anti-infective for systemic use (ATC-class J)	Doxycycline Amoxicillin Cefalexin Amoxicillin clavulanic acid Phenoxymethylpenicillin

According to the univariate analysis (Table 4) age and lung capacity (FVC% and DLCO/VA%) were predictors of initiating antifibrotic medication. Gender and the number of concomitant medications were not statistically significant predictors. In the multiple logistic regression analysis, the only independent predictor for initiating antifibrotic was the age at diagnosis (Table 5).

**Table 4:** Univariate logistic regression analysis for initiating antifibrotic treatment

Variable	OR	95% CI	*p*-value
Age 66–75 vs 50–65	0.66	0.32–1.35	0.25
Age over 75 vs 50–65	0.21	0.10–0-45	** < 0.0001**
Age 66–75 vs over 75	3.14	1.71–5.77	**0.0002**
Male vs female	1.40	0.82–2.36	0.21
FVC 75% or under vs FVC over 75%	2.29	1.27–4.14	**0.0061**
DLCO/VA 55% or under vs DLCO/VA over 55%	1.80	0.99–3.27	**0.05**
Other medicines 2–4 vs 0–1	1.04	0.47–2.28	0.92
Other medicines 5 or more vs 0–1	0.91	0.51–1.61	0.74
Other medicines 2–4 vs 5 or more	1.15	0.57–2.30	0.70

**Table 5:** Multiple logistic regression analysis for initiating an antifibrotic treatment

Variable	OR	95% CI	*p*-value
Age 66–75 vs 50–65	0.75	0.33–1.68	0.48
Age over 75 vs 50–65	0.19	0.08–0.48	**0.0004**
Age 66–75 vs over 75	3.96	1.83–8.55	**0.0005**
FVC 75% or under vs FVC over 75%	1.73	0.86–3.48	0.13
DLCO/VA 55% or under vs DLCO/VA over 55%	1.83	0.92–3.64	0.086

### Subgroup analysis: patients diagnosed on 1.6.2013 or after

For a subgroup analysis the study population was limited to patients diagnosed from 1.6.2013 onwards when the first antifibrotic treatment became available for IPF patients. The delay from the diagnosis to the initiation of the antifibrotic regimen was defined in 92 patients. The mean interval from diagnosis to the first purchase of the antifibrotic was 367 (SD 429, range 0–1632) days. The mean treatment duration was 633 days. Sixty-five percent of patients initiated the medication within a year. A comparison of the groups “Initiation ≤ 1 year” versus “Initiation > 1 year” is presented in Table 6. Twenty-five percent of patients whose FVC % predicted fulfilled the reimbursement criteria (50–80% or 50–90% of predicted) did not receive antifibrotic treatment (reason not known, insufficient data).

**Table 6:** Comparison of patient groups: treatment initiated ≤ 1 year versus > 1 year (patients diagnosed 1.6.2013 or after included). Data are presented as mean ± sd unless otherwise stated

Variable	Initiation ≤ 1 y (*N* = 60)	Initiation > 1 y (*N* = 32)	*p*-value
Age at diagnosis(years)	70.0 ± 6.1	68.6 ± 8.5	0.41
50–65 years N (%)	12 (21.1%)	7 (23.3%)	0.91
66–75 years N (%)	31 (54.4%)	15 (50.0%)	
> 75 years N (%)	14 (24.6%)	8 (26.7%)	
Gender			
Male N (%)	46 (76.7%)	20 (62.5%)	0.15
Female N (%)	14 (23.3%)	12 (37.5%)	
BMI (kg/m^2^) median	27.1(25.0, 30.6)	28.4(26.0,31.6)	0.36
FVC (% of predicted)	70.1 ± 12.4	84.0 ± 12.8	< 0.001
FVC ≤ 75% N (%)	24 (63.1%)	7 (23.3%)	0.001
FVC > 75% N (%)	14 (36.8%)	23 (76.7%)	
DLCO/VA/VA (% of predicted)	55.6 ± 14.6	60.1 ± 12.1	0.11
DLCO/VA ≤ 55 N (%)	18 (54.6%)	10 (35.7%)	0.20
DLCO/VA > 55 N (%)	15 (45.5%)	18 (64.3%)	
Smoking at diagnosis			0.13
Never smoker N (%)	20 (35.1%)	13 (41.9%)	
Current smoker N (%)	3 (5.3%)	5 (16.1%)	
Ex-smoker N (%)	34 (59.7%)	13 (41.9%)	
Number of medications at diagnosis (other than antifibrotic) median	6.0 (3.0,9.0)	6.0 (5.0,9.0)	0.96
0–1 medication N (%)	6 (10,0%)	3 (9.4%)	0.54
2–4 medications N (%)	13 (21.7%)	4 (12.5%)	
≥ 5 medications N (%)	41 (68.3%)	25 (78.1%)	

In the univariate logistic regression analysis (Table 7) a significant predictor for initiating antifibrotic treatment within a year was low forced vital capacity (FVC ≤ 75% vs FVC > 75%). DLCO/VA, age, gender, or the number of medications were not significant predictors. For the multiple logistic regression analysis variables age, FVC, and DLCO/VA were selected. The only independent factor for earlier initiation was low forced vital capacity Table 7.

**Table 7:** Univariate analysis logistic regression analysis for the delay of initiation of antifibrotic treatment (≤ 1 year vs > 1 year). Patients diagnosed 1.6.2013 or after included

Variable	OR	95% CI	*p*-value
Age 66–75 vs 50–65	0.83	0.27–2.54	0.74
Age over 75 vs 50–65	0.98	0.27–3.50	0.97
Age 66–75 vs over 75	0.85	0.29–2.46	0.76
Male vs female	0.51	0.20–1.29	0.15
FVC 75% or under vs FVC over 75%	5.63	1.93–16.46	**0.0016**
DLCO/VA over 55% vs DLCO/VA 55% or under	2.16	0.77–6.07	0.14
Other medicines 2–4 vs 0–1	0.62	0.10–3.66	0.59
Other medicines 5 or more vs 0–1	1.22	0.28–5.32	0.79
Other medicines 2–4 vs 5 or more	0.50	0.15–1.72	0.27

## Discussion

During the last few years, several national and multinational real-world projects have reported on the use of antifibrotics. In this study cohort, 50% of IPF patients used antifibrotic therapy. An earlier study by Pesonen et al. [14] showed that between 2014–2016 less than 30% of Finnish IPF patients were initiated on antifibrotics. In Sweden, 64% of 540 patients in the SwedishIPF registry enrolled between 2014–2020 received antifibrotics [15]. A European survey in 2016 revealed that in a selected group of countries, 60% of patients with confirmed IPF diagnosis were treated [6]. In the United States, data from the IPF-PRO registry showed that 70% of patients used antifibrotic regimens [16]. All these studies were designed differently, and unidentical recruitment protocols were used, thus comparison of the results is not reasonable.

Yet, a substantial number of patients (50% in our cohort) are not treated with specific antifbrotic therapy. The reasons for reluctance to start antifibrotic medication has been studied and discussed in recent surveys [6]. Possible obstacles to initiating treatment were a stable or mild disease, diagnostic uncertainty, reimbursement/availability issues, and worry about the adverse effects and the interactions of the antifibrotics.

According to our results, age was a predictor of treatment initiation, which is confirmed in several other real-world studies on IPF [14, 16, 17]. Patients in our cohort were older than in other similar studies [18, 19]. The results indicated a tendency treating under 75-year-old patients. However, Leuschner et al. [20], concluded in their study that the effect of antifibrotics were similar in older patients (≥ 75 years) as well as in younger age groups.

Early initiation of antifibrotics has been highlighted [21, 22]. In our study, the mean time gap from diagnosis to treatment initiation was 367 days, ranging from 0 to 1632 days. In addition, low forced vital capacity predicted earlier initiation of medication. Some of the treatment delays are explained by the Finnish reimbursement procedure. Processing of the reimbursement applications takes 2–6 weeks. Until 2015, the upper limit for reimbursement for FVC% was 80% which meant that once reached the markets, antifibrotic drugs were not an option for patients with FVC% over 80%. Even now the reimbursement limit (FVC % 50–90%) restricts early-stage initiation as physicians must wait for the FVC to decline to 90% of predicted or lower.

In our study, discontinuation rate at one year was 22.9% and 21.1% for pirfenidone and nintedanib respectively. In a single-center study from England [23] the discontinuation rate was 58% for pirfenidone and 53% for nintedanib at 18 months. More patients with pirfenidone had stopped than with nintedanib at 3 and 6 months but the difference disappeared at 18 months and the numbers of patients were small. An extensive registry study from United states, reported discontinuation of 10.6% and 10.7% nintedanib and pirfenidone users, respectively [24]. In our work, there was no evident difference in the persistence of the two products (Fig. 1). We do not know the reasons for the discontinuation, but side effects and lack of immediate response are the most likely. Nevertheless, recent study by Cilli et al. showed that elderly patients experience more adverse events during antifibrotic therapy than younger patients but they remain on therapy despite of that [25].

The therapeutic burden of ILD patients has been acknowledged [10]. Polypharmacy and complex medication regimen are common [26]. In addition, Khor et al. have postulated that concomitant medication burden is associated with intolerance of antifibrotic medications [27]. Our study confirms that patients have multiple medications already at the time of diagnosis. The used medications reflect comorbidities such as cardiovascular diseases, gastroesophageal reflux disease, and COPD.

Pain relievers such as paracetamol ± codeine and oxycodone were the second most used concomitant medications. This indicates the high need for symptom relief in IPF patients as these products are used for pain, cough, and dyspnoea [28]. The use of antibiotics was surprisingly high reflecting perhaps the susceptibility to respiratory infections or the tendency of respiratory doctors to treat for safety’s sake before the diagnosis is confirmed.

We acknowledge some weaknesses in the study. Firstly, there are limitations inherent to retrospective observational RWD studies. There is potential bias concerning missing data. Relatively small patient numbers were described in the nintedanib subgroup, which is a clear limitation and reflects the later approval of nintedanib for clinical use in Finland. Accordingly, direct comparisons between the two antifibrotics should be made judiciously. Medication use in this study was based on pharmacy dispensations, which do not account for the possible stockpiling of medicines. It was assumed that all medications dispensed were consumed and that the first day of consumption was the same day as the day of dispensation, which may not have been the case in all instances. This may have resulted in an overestimation of treatment persistence and duration.

However, we want to also highlight the strengths of the study. The study population consisted of confirmed IPF patients. For ethical reasons, only consented patients were included. The FinnishIPF project has been ongoing since 2012 offering long follow-up data for the medication use and disease course. In Finland, all permanent residents are entitled to public health care (regardless of their financial situation). Therefore, FinnishIPF data is globally unique, as it is not skewed for socioeconomic grounds. In addition, the Social Insurance Institution of Finland (SII) serves as a reliable data source of prescription medications.

## Conclusions

The use of antifibrotics has increased but still, many patients are untreated. In this study, younger age was a predictor of drug initiation, yet future studies should aim at evaluating which patients benefit most from the medications. The initiation of antifibrotics after diagnosis was slow, probably due to reimbursement limitations. Polypharmacy was common but it did not associate with initiating the antifibrotic therapy. The reasons for discontinuation of the antifibrotic therapy during the first year should be a focus in clinical work and further studies.

## Data Availability

The datasets generated and/or analyzed during the current study are not publicly available due to consent agreements but are available from the corresponding author on reasonable request.
